# Supplementary material for: Effect of inhibition indexed by auditory P300 on transmission of visual sensory information
Source: PLoS One. 2021 Feb 22;16(2):e0247416. doi: 10.1371/journal.pone.0247416 (PMC7899349; doi:10.1371/journal.pone.0247416)
Supplement: Attachment — Submitted filename: Chatroudi_et_al_review.pdf [file pone.0247416.s001.pdf]

On each trial an auditory odd-ball (or a standard) was presented 400ms prior to a visual odd-ball (or a standard). Participants counted the number of auditory odd-balls and quickly responded to each visual odd-ball. The auditory odd-balls generated the P300 ERPs at 300-500ms (all times are measured relative to the initial auditory onset), and slowed the subsequent responses to the visual odd-balls. The P300 ERPs to a visual odd-ball (650-900ms) was suppressed when an auditory odd-ball preceded the visual odd-ball. An auditory odd-ball reduced posterior alpha power at 300-500ms (prior to the onset of visual odd-ball at 400ms) and a visual odd-ball (when preceded by an auditory odd-ball) reduced posterior alpha power at 900-1300ms. In addition, the alpha power at 300-1000ms in the LORETA-defined posterior alpha sources was significantly correlated with the preceding P300-EEG (up to 500ms) in the LORETA-defined P300 sources (with no delayed correlations in the reversed direction). These are my understanding of the primary results. They appear to be reliable (given the statistics provided) and obtained with appropriate methods, thus are likely to contribute to the relevant literature. I personally think that the theoretical discussions are excessive given the scope of the results, but the authors should have the freedom to discuss them so long as speculative inferences are clearly indicated as such. Below are some comments and suggestions that the authors may interested in considering in their revision.

1. The authors talk about "the inhibitory effect of the P300," but P300 is a phenomenologically defined ERP component. Any inhibitory effect must be exerted by some underlying processes (e.g., auditory odd-ball detection, the updating of odd-ball counts in WM, etc.) that generate P300 recorded from the scalp.

2. The reference [25 (1991)] for the neural sources of alpha oscillations may be a bit dated. A study by Bollimunta et al. may be relevant (Bollimunta, Chen, Schroeder, and Ding, 2008, J Neurosci). Also, equating posterior alpha to sensory inhibition may be a bit simplistic. Posterior alpha oscillations have also been linked to oscillations of visual awareness (e.g., Mathewson and colleagues), the sequential structuring of incoming visual information (e.g., Jensen et al., 2014, Trends in Neurosciences), etc.

3. Lines 132 to 135. The two statements seem to be inconsistent. The first states that P300 predicts alpha ERD. The second, which is supposed to confirm the directionality of the first statement, states that information flows from the alpha ERD sources to the P300 sources.

4. Line 272. What is the "length" of a Morlet wavelet? It is Gaussian-windowed so would its range not be infinite? The authors might provide the temporal standard deviation instead.

5. The permutation-based statistical testing described in lines 308+ uses the 99.5th percentile criterion whereas that described in lines 397+ uses the 95th percentile criterion. Is one of them a typo, or was there a rationale behind using different criteria?

6. In Figure 3, it is unclear to me what ROI is indicated by the red square.

7. Additional discussions on the Granger analysis may be helpful. First, the authors should NOT equate significant Granger values with causality. It is just a correlation analysis with a series of temporal delays (10ms increment in the study) within a time window (500ms in the study). For instance, a significant Granger value from P300 to alpha ERD just means that up to 500ms prior P300 EEG (with appropriate weighting of different prior time points) predicts the current alpha ERD. Significant Granger values are consistent with the interpretation that P300 is directly causing alpha ERD. However, it is also consistent with an alternative interpretation that some processes related to auditory odd-ball detection generate BOTH P300 and alpha ERD, but the latter with a longer delay, which could make Granger values from P300 to alpha ERD significant. That is, significant Granger values are consistent with causality/connectivity, but they do NOT imply causality/connectivity. I think that it is very important to respect this distinction.

8. To make the case that the Granger effect from P300 to ERD is uniquely in the alpha range, it might be useful to show the frequency dependence of the Granger value (to show that it peaks at alpha).

9. I do not understand how the time-frequency power (dB) plots shown in Figure 7 are related to the Granger values. Are the power values weighted by a Granger index (e.g., the logarithm of the ratio of the univariate error variance to the bivariate error variance)? Some details would be appreciated.

10. The asymmetry of Granger results between ERD -> P300 and P300 -> ERD is emphasized. However, is it not the case that Granger predictions could not go in the direction of ERD to P300 by design? If I understood the study correctly, the auditory stimuli always preceded the visual stimuli and the auditory-evoked P300 peaked prior to the visual onset. That is, the auditory-evoked P300 preceded the visually-induced posterior alpha ERD. Then, how could the latter causally influence the former? A clarification would be appreciated.
